# Impact of Standardized Patient Programs on Pre-clinical Medical Students' Clinical Readiness: A Narrative Review of the Last Two Decades

**DOI:** 10.7759/cureus.92419

**Published:** 2025-09-16

**Authors:** Cristian V Toma, Aida Petca, Ioana G Visan, Alexandra Munteanu, Alexandru Ciudin, Viorel Jinga

**Affiliations:** 1 Department of Urology, Carol Davila University of Medicine and Pharmacy, Bucharest, ROU; 2 Department of Urology, Professor Doctor Theodor Burghele Clinical Hospital, Bucharest, ROU; 3 Department of Obstetrics and Gynaecology, Carol Davila University of Medicine and Pharmacy, Bucharest, ROU; 4 Department of Obstetrics and Gynaecology, Elias Emergency University Hospital, Bucharest, ROU; 5 Department of Medical Simulation, Carol Davila University of Medicine and Pharmacy, Bucharest, ROU; 6 Department of Urology, Hospital Universitari de Mollet, Barcelona, ESP; 7 Department of Urology, The Academy of Romanian Scientists, Bucharest, ROU

**Keywords:** educational tool, medical education, medical simulation, pre-clinical medical students, standardized patient

## Abstract

Standardized patients are a valuable educational tool for students, residents, and even professionals in the healthcare domain. Although their role in improving quality of care, clinical skills, and confidence is well determined for senior students or residents during their formation years, when it comes to pre-clinical year students, simulated patient encounters have a less well-determined role and curriculum integration. With regard to medical education, pre-clinical years are traditionally marked by core knowledge and theoretical lectures conventionally taught in lecture halls, with minimal or no patient contact, given the focus in this vital period of medical formation on gathering information and learning basic science topics. Given the fact that many universities have introduced simulated patient encounters for students in their early medical years, the purpose of this article is to review and describe such experiences published and found in medical databases to fully grasp the benefits, the limitations, and the future directions of such simulated educational activities for young students. Although medical knowledge is scarce and clinical skills are yet to be formed, literature articles exhibit how important social and psychological aspects of doctor-patient encounters can be addressed and rehearsed early on, in controlled, simulated environments that permit exercising verbal and non-verbal communication, appropriate posture, and attitude towards patients in an immersive setting, hopefully improving overall quality of care. In conclusion, the studies described successful models for implementing such simulated activities in the medical curriculum, with positive outcomes and feedback from participating students and educators.

## Introduction and background

Originally developed in the 1960s by Barrows as a personal tool for neurological teaching and assessment, standardized patients later became an essential part of medical education, improving curricular development through various programs [1]. In simple terms, a standardized patient is an individual carefully trained to portray the symptoms, history, and behavior of a real patient in a consistent manner, ensuring that every student who encounters them has a uniform learning experience. As a founding father of this educational instrument, Barrows believed that standardized patients may very well exceed the value of real patients in teaching medical trainees, as they can be used to evaluate a greater number of students or participants and can offer a wider array of challenges to be handled [1].

Since their initial introduction in the medical education field, standardized patient programs have evolved significantly over the last 25 years [2]. Some authors consider that the future of standardized patient courses will include maturation of current projects and the emergence of new and highly specialized ones (field-oriented, method-based, or occupation-related) [2].

According to Barrows and Tamblyn, medical education would benefit from changes in the curriculum. That includes student-centered approaches and small-group learning methods as pertinent educational goals for problem-based learning, which is the learning process that occurs when work is focused on solving or understanding a problem [3].

Medical training aims to equip all physicians with the necessary knowledge and abilities to provide the best patient care with professionalism and improved patient outcomes and is the final goal of all medical education programs [4].

Comparing the effectiveness of traditional teaching methods with newer ones based on simulation with deliberate practice, existing literature favors outcomes of the latter, with a considerable body of evidence suggesting improved patient care outcomes and demonstrating superiority when it comes to skills acquisition in various medical domains [4].

There has been an increasing interest in early clinical exposure for medical students as many members of the medical education community drew attention to the importance of this experience early on in medical formation to help form skills and cognition to put together for later medical practice [5]. Some educators believe that the traditional curriculum, with its lack of integration, has led to underachievement in learning and a poor student impression regarding the relevance of current approaches for upcoming healthcare practices [6].

Given the fact that data is scarce regarding simulated activities with standardized patients for novices and teaching methods have evolved and changed over time, our paper was developed with the purpose of retrieving and detailing in a descriptive manner as many articles as possible on this topic in order to verify areas in which standardized patients were used successfully for pre-clinical medical students.

## Review

Materials and methods

We conducted a literature search of four medical databases: Web of Science, ScienceDirect, PubMed, and Scopus for all papers describing experience with standardized patients in teaching pre-clinical medical students or any simulated patient encounter (peers, actors, or faculty members) for educational purposes during the pre-clinical stage.

Using the keywords "standardized patient/simulated patient" and "pre-clinical medical students", "medical education", "actors", and "medical simulation", a total of 5,074 papers were found.

The article's inclusion criteria were limited to the years 2004-2024, required English language, provided full-paper access, and targeted medical students in pre-clinical years. Articles comprising student and/or faculty perception of the didactic activity were included.

**Figure 1:**
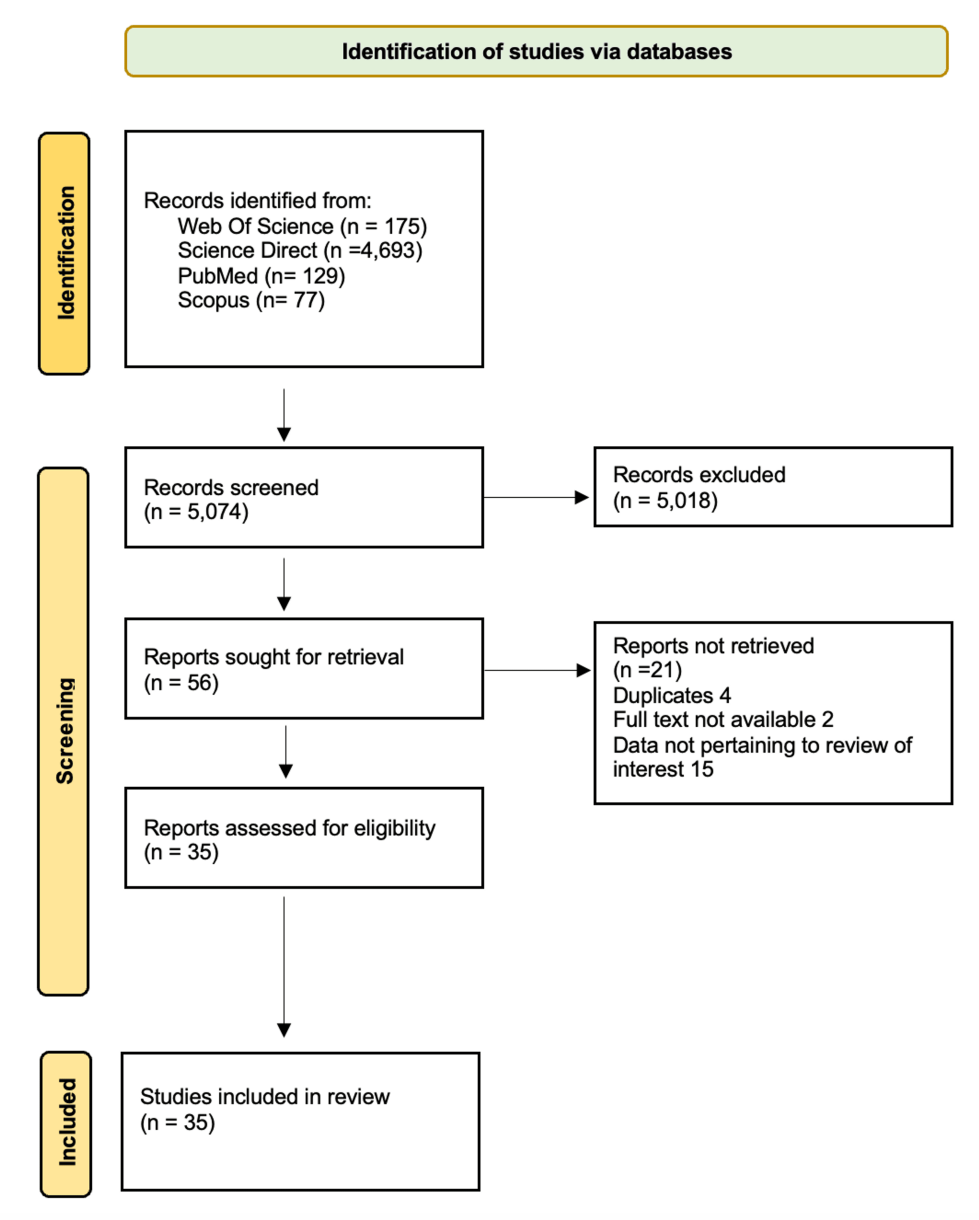
Search strategy

Exclusion criteria were articles regarding experiences for nursing, dentistry, or pharmacy students or residents; articles describing educational encounters for medical students during their clinical rotations; or articles about healthcare professionals. Papers describing sessions for pre-clinical medical students using simulation and simulated scenarios with mannequins were also excluded from this review. After all articles found were screened, a total of 55 articles were included for full-text review for this paper, of which 35 were included for this literature review. The screening process is illustrated in Figure 1.

Results

Reviewed papers in our literature research found many uses for standardized patients in pre-clinical medical training, with a greater number of articles describing experiences and interventions using simulated scenarios for first-year medical students (n = 14, see Table 1 for study details) compared with articles found with second-year students (n = 17, see Table 2 for study details). Two articles describe experiences with both first- and second-year students, one article relates interventions for pre-clinical third-year students, and one does not specify which pre-clinical year (see Table 3 for study details).

**Table 1:** Summarized articles with SP experience for first-year medical students SP: Standardized Patient; OSCE: Objective Structured Clinical Examination

No.	Year	Article	Authors	Number of Students	Topic	Conclusions	Study Type
1.	2003	The BELIEF Instrument: A Preclinical Teaching Tool To Elicit Patients’ Health Beliefs [7]	Alison E. Dobbie, MD, et al.	197 first-year medical students	Cultural interviewing tool	Useful tool for teaching interviewing skills to preclinical medical students	Pilot intervention study
2.	2020	Motivational Interviewing Training: A Case-Based Curriculum for Preclinical Medical Students [8]	N. Nicole Jacobs, PhD, et al.	68 first-year medical students	Teaching students early preventative medical care, disease prevention, and lifestyle modification through patient involvement	Training was effective in teaching students the necessary skills in order to perform this technique in a wide range of cases	Quasi-experimental pilot study
3.	2021	A task-based learning strategy in preclinical medical education [9]	Roopashree Shenoy et al.	250 first-year medical students in 3 consecutive academic years (total 750)	Comparing tutorial-based conventional teaching methods with task-based learning strategies	Study results indicate that task-based learning provides a better understanding of certain topics in preclinical training	Quasi-experimental study
4.	2022	Evaluation of Preclinical Task-Based Learning Program in Medical Education [10]	Roopashree Shenoy et al.	49 first-year medical students in 8 focus groups	Early clinical exposure through task-based learning	Students believed there were many positive attributes to this teaching module	Qualitative research study
5.	2016	A simulation-based curriculum to introduce key teamwork principles to entering medical students [11]	Arna Banerjee et al.	103 first-year medical students split into 10 groups	Teaching basic teamwork and interpersonal skills	The course is highly regarded by faculty and trainees and has improved the preclinical curriculum	Quasi-experimental intervention study
6.	2007	Long-term Retention of Smoking Cessation Counseling Skills Learned in the First Year of Medical School [12]	Lynn Y. Kosowicz, MD, et al.	2 cohorts of first-year medical students (76 +75) followed through their 4^th^ year of training	Retention of smoking cessation counseling skills	Skills were well retained into the fourth year of medical education, with minimal reinforcement in the third year	Retrospective study
7.	2022	Transformative Learning and Critical Consciousness: A Model for Preclerkship Medical School Substance Use Disorder Education [13]	Andrew Muzyk et al.	122 first-year medical students	Teaching preclinical students to provide appropriate care for patients with substance use disorders	Students grew their critical consciousness towards patients with SUD and gained a better understanding of this topic	Qualitative study
8.	2020	The Simulated Virology Clinic: A Standardized Patient Exercise for Preclinical Medical Students Supporting Basic and Clinical Science Integration [14]	Jennifer M. Jackson, MD, et al.	278 first-year medical students	SP cases portraying viral infections for knowledge integration and problem-solving practice	Simulation proved to be an effective method to teach and help students to integrate knowledge in a realistic context	Quasi-experimental pilot study
9.	2022	Antiviral Pharmacology: A Standardized Patient Case for Preclinical Medical Students [15]	Michael K. Jones et al.	144 (in 2020) and 145 (in 2021) first-year medical students	Correlating and integrating pharmacology knowledge with clinical context	Practice was very well received by learners and was perceived as an appropriate approach for integrating knowledge	Quasi-experimental educational intervention study
10.	2021	Randomised controlled monocentric trial to compare the impact of using professional actors or peers for communication training in a competency-based inverted biochemistry classroom in preclinical medical education [16]	Achim Schneider, David et al.	155 first-year medical students	Awareness enhancement of biochemistry knowledge clinical relevance using role plays with peers and standardized patients	Role-playing exercises with both peers and SPs helped improve students' self-perceived competency	Randomized controlled study
11.	2020	“I Have a Cough”: An Interactive Virtual Respiratory Case-Based Module [17]	Nelia Afonso, MD, et al.	122 first-year medical students	Using virtual resources to teach the respiratory physical exam for students in preclinical years	Virtual teaching instruments can be a valuable substitute for conventional methods and help improve telemedicine consultation skills, clinical correlation, differential diagnosis, and interpretation of physical exam findings	Quasi-experimental pilot study
12.	2024	Comparing Peer-Taught Student Tutors to Faculty-Taught Student Tutors in Educating Medical Students on Musculoskeletal Ultrasound [18]	Matthew Aquino et al.	60 first-year medical students	Teaching musculoskeletal ultrasound concepts using standardized patients and peers or ultrasound instructors	The ability of first-year medical students to learn basic ultrasound notions is independent of the type of tutor chosen	Quasi-experimental, comparative study
13.	2023	Comparing the Perceptions of Reciprocal- and Near-Peer Objective Structured Clinical Examinations (OSCEs) in Medical Students [19]	Olivia Calisi et al.	135 (first session) and 129 (second session) first-year medical students	Virtual practice for OSCE using peers for simulated cases and feedback	Students considered near-peer practice as more beneficial than reciprocal-peer experiences	Quasi-experimental study
14.	2017	Correlating Preclinical Ambulatory Care Specialty Exposure with First-Year Medical Student Performance on an Objective Structured Clinical Examination [20]	John E. Nolan III et al.	197 first-year medical students	Comparison of OSCE scores obtained by students with ambulatory specialty exposure	Results suggest that preceptors from a variety of specialties can be used for preclinical ambulatory exposure for preclinical medical training	Retrospective cohort study

**Table 2:** Summarized articles with SP experience for second-year medical students SP: Standardized Patient

No.	Year	Article	Authors	Number of Students	Topic	Conclusions	Study type
1.	2014	From board to bedside—training the communication competencies of medical students with role-plays [21]	Katharina Luttenberger et al.	182 second-year medical students	Role-playing for communication skill improvement	Students perceived the course as very helpful, and the authors believe it can be easily introduced in the curriculum without significant additional costs	Quasi-experimental study
2.	2014	Evaluating the short-term effects of a communication skills program for preclinical medical students [22]	Young-Mee Lee et al.	111 second-year medical students	Testing the effects of a communication skills course on preclinical medical students	Developed courses proved to be very beneficial, even if taken for a short period of time	Quasi-experimental study
3.	2022	Vaccine Hesitancy Counseling—An Educational Intervention to Teach A Critical Skill to Preclinical Medical Students [23]	Arati Kelekar et al.	126 second-year medical students	Teaching counseling skills using standardized patients as vaccine-hesitant patients	Simulated scenarios proved effective in teaching students the necessary skills to improve communication	Quasi-experimental study
4.	2022	Interviewing teen parents: Simulated Patient Experience for Clinical Education and Outreach [24]	Peter Averkiou et al.	64 second-year medical students	Using simulation-based education to teach preclinical students history-taking skills and to provide early clinical exposure	Medical students found the session very helpful in practicing history-taking skills for pediatric caregivers	Quasi-experimental intervention study
5.	2022	A structured approach to shared decision-making training and assessment of knowledge, attitudes and perceptions of second-year medical students [25]	Charlotte Leblang et al.	103 second-year medical students	Training and exercise for shared decision-making for preclinical medical students	Students felt more comfortable and knowledgeable with shared decision-making and considered having a greater motivation using the taught skills in their future careers	Quasi-experimental intervention study
6.	2018	Implementation of a standardized patient program using local resources in Avalon School of Medicine [26]	Jesse Ramey et al.	24 second-year medical students	Determining efficiency for SP programs	The study showed increased students’ performance regarding clinical abilities	Quasi-experimental study
7.	2018	Teaching diagnostic reasoning: using simulation and mixed practice to build competence [27]	Heather Murray, MD, et al.	50 second-year medical students	Emergency department simulations to teach clinical reasoning	Students found the sessions as very helpful in their clinical rotations, as documented by follow-up after 18 months	Quasi-experimental intervention study
8.	2020	Evaluation of the effect of a new clinical reasoning curriculum in a pre-clerkship clinical skills course [28]	Arati Kelekar et al.	101 second-year medical students	Introducing a preclinical clinical reasoning curriculum	The study confirms that the said curriculum provides students with the necessary training to navigate relevant symptoms	Quasi-experimental study
9.	2020	Electroconvulsive Therapy: A Video-Based Educational Resource Using Standardized Patients [29]	Brandon Kitay et al.	63 second-year medical students	Using videos with standardized patients undergoing the necessary phases for ECT to teach and reduce stigma	The teaching method provides advantages compared to traditional methods, and student surveys showed improvements in viewpoints and knowledge	Quasi-experimental study
10.	2021	Tutors’ Perceptions of the Transition to Video and Simulated Patients in Pre-clinical Psychiatry Training [30]	Mitesh Patel et al.	45 groups of 5-7 second-year medical students each	Transitioning from in-person teaching to virtual sessions using standardized patients from the teacher’s perspective	The study showed that tutors were able to favorably transition with relative ease	Cross-sectional survey design
11.	2018	Development and validation of simulated virtual patients to impart early clinical exposure in endocrine physiology [31]	Akriti Gupta et al.	40 second-year medical students	Teaching endocrine physiology using virtual simulated patients	Created patients were very well received by students, and can be a feasible teaching method	Quasi-experimental pilot study
12.	2021	Simulation: An Innovative Approach to Engaging Preclinical Medical Students with Bioethics [32]	Christine E. Bishop et al.	140 second-year medical students	Teaching bioethical principles using simulated scenarios	Students found the event to be very beneficial for their future roles	Quasi-experimental intervention study
13.	2022	Integrating the Electronic Health Record into Patient Encounters: An Introductory Standardized Patient Exercise for Preclinical Medical Students [33]	Joseph A. Cristiano, MD, et al.	289 second-year medical students	Familiarizing students with EHR through simulated encounters with SP	The session was found to be very helpful in aiding students with incorporating EHR into patient encounters	Quasi-experimental intervention study
14.	2010	DEVELOPMENTS: Faculty, Students, and Actors as Standardized Patients: Expanding Opportunities for Performance Assessment [34]	Brian Mavis et al.	313 second-year medical students	Determining the impact of different types of standardized patients on students’ opinions	The choice of SP type should be based on the goals of the simulated encounter	Quasi-experimental study
15.	2012	Use of Breast Simulators Compared with Standardized Patients in Teaching the Clinical Breast Examination to Medical Students [35]	Jane R. Schubart, PhD, et al.	141 second-year medical students	Comparing teaching methods for clinical breast examination teaching methods	Evidence supports the use of simulators in teaching CBE, with no evidence proving that standardized patients are a more beneficial educational tool	Quasi-experimental control study
16.	2016	Using standardized patients versus video cases for representing clinical problems in problem-based learning [36]	Bo Young Yoon et al.	99 second-year medical students	Comparing learning experiences for problem-based learning	Standardized patients proved to be more effective than video cases for preclinical students	Quasi-experimental crossover study
17.	2014	Combining simulated patients and simulators: pilot study of hybrid simulation in teaching cardiac auscultation [37]	Hendrik Friederichs et al.	143 second-year medical students	Testing hybrid methods for teaching the basics of a clinical examination	Study results prove high acceptance of this hybrid teaching method and positive responses from students, tutors, and simulated patients	Quasi-experimental intervention study

**Table 3:** Summarized articles with SP experience for first- and second-year, third pre-clinical medical year, and not specified year of education SP: Standardized Patient

No.	Year	Article	Authors	Number of Students	Topic	Conclusions	Study Type
1.	2023	Early Exposure to Lesbian, Gay, Bisexual, Transgender, Queer (LGBTQ+) Medicine: Assessing Confidence and Comfort in Preclinical Medical Students [38]	Kelsee K. Zajac et al.	25 first- and second-year medical students	Raising confidence and comfortability for students when dealing with LGBTQ+ topics through interactive workshops	Study results suggest that such developed workshops can significantly improve confidence and comfort for preclinical medical students and can lead to improvement in patient care	Quasi-experimental study
2.	2012	Integrating Lesbian, Gay, Bisexual, and Transgender (LGBT) Content into Undergraduate Medical School Curricula: A Qualitative Study [39]	Gina M. Sequeira et al.	Preclinical (not specified)	Familiarizing preclinical medical students with LGBT content and	LGBT health educational sessions were viewed as meaningful and helpful by students	Qualitative pilot study
3.	2021	Early Intervention for LGBTQ Health: A 10-Hour Curriculum for Preclinical Health Professions Students [40]	Matthew S. Minturn et al.	40 first- and second-year medical students	Preparing students to care for LGBTQ patients with lectures, discussions and standardized patient encounters	Intervention proved to be beneficial regarding students’ confidence but less effective in increasing LGBTQ-related medical knowledge	Quasi-experimental study
4.	2015	Helping medical students to acquire a deeper understanding of truth-telling [41]	Samia A. Hurst et al.	120 (2004) and 105 (2005) third-year medical students followed through their fifth year	Breaking bad news and teaching students the ethical aspects of truth-telling	Overall, the session helped students with feelings of comfort and competence regarding truth-telling	Longitudinal quasi-experimental study

Discussion

Standardized Patient Encounters for Improving Communication

Out of the 14 papers that describe simulated encounters with standardized patients for educational purposes for first-year medical students, most focus on stimulating and developing basic but fundamental skills for medical formation: communication, teamwork, interviewing abilities, and integrating knowledge. Some articles focus on communication skills only, emphasizing the importance of the medical interview for the doctor-patient relationship and the early practice during the medical formation years for such skills [7,8].

Shifting attention to another way of delivering educational content, two articles showed results obtained with task-based learning techniques for novices in medical training [9,10]. The outcomes showed improvement of knowledge, skills, and attitudes and students' impressions regarding the intervention and its relevance for their professional formation [10].

Other papers found and reviewed focused on challenging doctor-patient encounters. In a paper written by Luttenberger et al., the results show that students valued interactive didactic methods for teaching communication skills, with many of them considering that practicing the role of the physician was more important than that of the patient [21]. Almost all students showed interest in learning to communicate in difficult situations, and the majority of students declared being satisfied with the course and having their expectations met. Measurements were made with self-report questionnaires that had both a quantitative and a qualitative section and were analyzed with descriptive statistics [21].

In another article that evaluated the effects of communication skills interventions, the results showed an increase in self-perceived ability to communicate in all categories of communication tasks: introduction, building relationships, obtaining information, understanding the patient's point of view, and closing the dialogue. The questionnaire used was a 25-item form that included building relationships, initiating the session, gathering information, understanding perspectives, and providing closure. All participants were asked to rate each item on the form using two separate five-point response scales: one regarding the perceived level of importance and the other regarding competence [22].

In an additional article that tackled the issue of communication and improving communication skills in second-year medical students, the results show a significant increase in students' self-perceived confidence and knowledge regarding recommending and counseling patients hesitant to vaccinations [23].

An interesting exercise for practicing communication skills using standardized patients is described by Averkiou et al., with outcomes proving that the activity was very well received by participants, and students believed this session was relevant to their future careers [24].

Another important aspect of the doctor-patient relationship is shared decision-making, which can also be learned and practiced through better communication skills. For such abilities in second-year medical students, Leblang et al. describe an educational intervention through which second-year medical students would engage and practice shared decision-making with standardized patients. The results pointed out that students believed that they felt more knowledgeable, more comfortable, and more motivated to use this approach in the future. The authors note that shared decision-making helps improve patient engagement in healthcare decisions and quality of care [25].

Standardized Patient Sessions for Improving Teamwork

On another note, other authors focused on improving or developing teamwork principles for first-year medical students [11]. Barnejee et al. describe a one-day immersive simulated activity designed to facilitate these principles based on six teamwork exercises for 420 first-year medical students (103 in 2007, 105 in 2008, 111 in 2009, and 101 in 2010). Participants were divided into groups, with all students participating in all six 75-minute exercises (of which one included a standardized patient encounter). At the end of the day-long course, participating students were requested to evaluate each exercise, the team facilitator, and the overall session development. The study shows how exercises have changed and improved over the years to better serve students' needs and accommodate the desired teaching curriculum. Analyzing the data obtained from day-long session evaluations, the authors conclude that what they created is highly beneficial for any medical school that intends to introduce teamwork exercises into the curriculum, and that participating students highly regarded these activities [11]. Such educational interventions attract attention to the fact that helping medical professionals improve teamwork principles has a patient-care quality impact.

Standardized Patients Used for Practicing Clinical Reasoning

Besides communication skills being the most frequent topic when using standardized patients as educational tools for pre-clinical medical years, clinical reasoning was also found to be an important subject for simulated activities [26-28]. According to Murray et al., simulated activities with standardized patients can be successfully used to teach second-year medical students diagnostic reasoning regarding emergency medicine, with the help of experienced emergency physicians, realistic environments, and feedback. Relying on standardized patients for this activity, students were exposed to three cases portraying an acute care presentation and were encouraged by physicians to identify key features for the presented cases. The program was rated by participants immediately after the session and after 18 months, with results showing that students appreciated the activity and considered it memorable in the long term [27].

Described by Kelekar and Afonso, another simulated course for second-year medical students presents a successful model for implementing a clinical reasoning curriculum using standardized patients and faculty-guided activities. In this study, the results obtained at OSCE (Objective Structured Clinical Examination) by participants in the simulated didactic activity (new clinical reasoning curriculum, three sessions of a total of six hours) were compared to those of second-year medical students from the previous year (comparison cohort with no clinical reasoning curriculum). The results obtained after the evaluation of study cohort students demonstrated benefits in improving clinical differential diagnosis skills for the clinical reasoning curriculum and motivated authors to consider expanding the curriculum for M1 students [28].

In another study, the authors narrate their experience introducing a standardized patient program to 24 pre-clinical medical students [26]. In this case, standardized patients were used to enhance the learning experience for students and fill in the gaps where necessary. Instructors were used to guide, offer feedback, and adjust the standardized patient encounters according to students' needs, and clinical physicians participated in the activities to grade students during examinations. The results showed that introducing the program led to an improvement in students' grading. The authors draw attention to the fact that current clinical medicine focuses on patient safety and quality of care, and such aspects should be kept in mind when organizing educational programs for future doctors [26].

Standardized Patient Sessions Regarding LGBTQ+ Health-Related Aspects

Studies tackling educational interventions for young students regarding LGBTQ+ health show that such activities can help students with confidence and comfort when assessing patients [38] and that these interventions are considered valuable for students' future careers [39].

In a paper by Zajac et al., referring to students' confidence and comfort while assessing patients from the LGBTQ+ community, 25 first- and second-year pre-clinical medical students participated in an interactive two-hour workshop consisting of simulated patient encounters, discussions, seminars, and debriefing. The authors mention that all participants in this workshop (standardized patients and facilitators) were members of the LGBTQ+ community. Consenting students were asked to complete a pre- and post-workshop survey regarding demographic information and LGBTQ+ healthcare information. The survey included basic demographic questions (gender identity, year in medical school, ethnicity/race, and sexual orientation) and 16 specific LGBTQ+ healthcare statements that were answered using a seven-point Likert scale (confidence level regarding self-education, using proper terminology, using proper pronouns, terminology, process and consent challenges, etc.). The results obtained through this questionnaire showed that students found the intervention helpful in increasing their confidence and comfort when assessing members of the LGBTQ+ community. The authors point out that such educational interventions can produce superior quality of care over time [38].

Another article describes a 10-hour course for pre-clinical medical students as an early intervention for LGBTQ+ health [40]. This study presents results obtained after an educational intervention with 40 first- and second-year medical students who participated in a 10-hour curriculum consisting of lectures, small-group discussions, terminology, history-taking, primary care, transition care, and practice sessions with standardized patients. The course was structured in five two-hour sessions, with the fifth session's first hour consisting of three standardized patient cases through which students rotated in small groups (for a less intimidating experience), followed by a debriefing session. Pre- and post-course surveys were completed by students using a Likert scale regarding self-confidence and knowledge acquisition (participant feels equipped to sensitively/effectively elicit information about sexual behavior, sensitively/effectively elicit information about sex anatomy and gender identity, articulate health needs for LGB patients, articulate health needs for transgender patients, summarize primary care recommendations for LGB patients, summarize primary care recommendations for transgender patients, and identify resources in the community for LGBT patients). The results showed that students considered that the experience significantly increased their self-confidence regarding assessing patients from the LGBTQ+ community but with poorer outcomes in the knowledge acquisition aspect, with authors considering that this may be related to the few medical knowledge that students have at this level of medical formation and the suboptimal question format [40].

Another intervention in the medical education curriculum related to LGBT content is described by Sequeira et al. in a paper describing an optional course consisting of three one-hour didactic sessions and one standardized patient experience for pre-clinical medical students, followed by completing an electronic feedback form. Herein, the authors conclude, based on student feedback, that such educational interventions are highly valued by students who considered the chosen content relevant to their future careers and acknowledged the current lack of exposure to LGBT-related care issues [39].

Standardized Patient Sessions Used in Addiction Medicine

Of all aspects considered in this review, the most interesting findings were regarding educational activities that included teaching counseling skills but also assessing retention by following students through their fourth year of medical education (with brief reinforcement in their third year) [12].

Kosowicz et al. describe how two cohorts of students were instructed through didactic sessions, small group discussions, and standardized patient encounters to provide adequate smoking cessation counseling. Students included in this study were pre-clinical but were followed through for knowledge acquisition in their clinical rotation years. During their third-year Family Medicine ambulatory rotation, the same students were motivated to identify smoking as an important cardiovascular risk factor and perform a short in-office counseling intervention [12].

The total time allocated for this didactic intervention was five hours, with four of them in the first year and one hour in the third year. During their fourth-year assessment, students had to perform 14 standardized patient encounters, of which one was specifically trained to require smoking cessation counseling skills. According to the authors, this study is one of the first to assess knowledge and skill retention over time, with results showing that information learned and practiced through simulation was well retained in the following years. The study encourages the teaching and reinforcement of smoking cessation counseling skills and proves that the retention rates of the skills taught are important [12].

Besides smoking cessation counseling, another study found that standardized patients and simulated patient encounters can be used for teaching first-year medical students about substance use disorders. This article focuses on activities based on the transformative learning method and improving critical consciousness through lectures, workshops, and standardized patient encounters. During the academic year 2021-2022, first-year medical students engaged in cognitive, affective, and psychomotor learning activities. Besides a mixture of didactic activities (a total of 12 lectures, two workshops, team-based learning exercises, and opioid overdose simulation), students had the opportunity to perform motivational interviewing with standardized patients (counseling, feedback from patients, and self and peer evaluation). As part of the module, students were asked to write an essay as a reflection regarding their personal beliefs about treating patients with substance use disorders, and 30 random papers (25% of all papers) were chosen for qualitative analysis from this cohort. This analysis revealed that students' contemplative writing exposed hostile attitudes towards patients with substance use disorder prior to realistic encounters and the use of metonymy. The authors conclude that early and frequent presentation of patients with substance use disorders to students can help improve empathy and soften students' perception regarding this population group, with an early exposure allowing entering clerkship years with a lower sense of discredit [13].

Standardized Patient Encounters in Specific Medical Disciplines

Some papers describe experience with first-year medical students who require applying medical knowledge appropriate to their formation level.

Domains such as virology [14], pharmacology [15], biochemistry [16], psychiatry [29,30], endocrinology [31], and ethics [32] tackle with using standardized patients in an innovative way to deliver educational content, with presented results showing that students found these activities very helpful [14], that they were very well received by students [15], that students benefit from such early communication skill exercises [16], that the didactic content and means of delivery were appreciated [29], that students were permitted to learn and practice at their own pace [31], and that the intervention was relevant to their future careers [32].

Out of all the articles searched and selected, only one paper was included describing simulated encounters for pre-clinical third-year medical students [41], in which authors conclude that a truthful educational intervention was found to be successful in introducing students to the issues related to truth-telling in the medical practice with applicability earlier and later in their medical formation years in improving communication skills and emotional aspects of ethical considerations (as students progressed, their focus shifted from a personal to a relational point of view) [41].

Standardized Patient Sessions to Improve Physical Examination

Regarding the topic of integrating theoretical notions according to the level of training, Afonso et al. describe experience with online interactive sessions using simulation to teach pre-clinical medical students the respiratory physical exam and communication skills through telemedicine standardized patient interviews. In this study, 122 first-year medical students participated in a course that included a demonstration of the respiratory physical exam delivered via videoconference, a telemedicine consultation for fever and cough with a standardized patient, and a final part for case discussions. Out of the 122 students who participated in this session, 57 (47%) students completed a voluntary survey at the end of the course, with the majority of students believing the session helped them with telemedicine communication skills (93%) and notions on performing a respiratory physical exam (84%) [28].

Standardized Patients Used for Introducing Ultrasound Notions

We bring to your attention a paper that presents an educational intervention regarding the teaching of musculoskeletal ultrasound to first-year medical students using standardized patients and peer-taught student tutors, compared with faculty-taught student tutors [18]. In this article, Aquino et al. mention that 60 first-year medical students participated in the study and were asked to identify volar forearm anatomic structures using ultrasound on standardized patients, guided by ultrasound instructors or peer-taught student tutors. Although the study focuses primarily on educational outcomes using different types of tutors, this paper shows that standardized patients can be used for various teaching events, even early on during medical formation. Participating students were split into four teaching groups and randomly assigned to a UI or PTST. A standardized patient was available for each group, providing their right forearm for teaching and assessing. Workshops consisted of a 30-minute introduction, instruction, and demonstration session, followed by a student illustration of retained information on standardized patients without assistance or feedback. Next, students had to complete a mandatory survey to assess confidence regarding identifying anatomical structures and ultrasound technique at the end of the session. The study results show that there was no statistical difference between the two teaching groups in accurately identifying anatomical structures, with more students from the ultrasound instructor group declaring feeling "very confident" in explaining taught ultrasound concepts [18].

Standardized Patients Used for OSCE Practice

Given their importance in students' assessment during their medical formation, this paper also discusses educational interventions, OSCE-related, using standardized patients for first-year medical students [19,20].

In a paper written by Calisi et al., the authors were interested in studying the effects of pairing first-year medical students with either near-peers (fourth-year medical students) or reciprocal peers for one week (with students paired with reciprocal peers also acting as standardized patients and switching roles). The purpose of this intervention was to check improvements in OSCE skills. The results showed that participating in the reciprocal-peer group and having the opportunity to act as patients and graders for other students was considered an advantage for the participants, while working with near-peer students was considered more valuable through the provided feedback, offering an increased level of perceived confidence regarding clinical skills [19].

Also OSCE-related, another article presents results after correlating different ambulatory specialty exposures for first-year medical students and students' performance on OSCEs [20]. In this paper, Nolan et al. describe outcomes following an educational intervention consisting of exposing 197 first-year medical students to one out of 10 outpatient specialties, followed by OSCE grading (communication, history-taking skills, and elicitation of patient concerns). Based on students' results obtained after OSCE assessments, the authors conclude that overall case and examination scores did not vary so much between students who participated in the generalist or specialist preceptor group, but they did notice that students who were exposed to outpatient settings alongside a generalist preceptor performed better at eliciting patient concerns during their first case [20].

Standardized Patients Used for Familiarizing With Health Records

On a different note, one paper describes an innovative use for standardized patients when introduced for teaching pre-clinical medical students. In this study, Cristiano et al. describe an intervention designed to familiarize students at an early level with patients and their electronic health records. The authors describe using notes, training videos, four standardized patient cases, and a simulated electronic health record for 289 second-year medical students who had the opportunity to practice patient encounters and interactions with electronic health records. Of all participating students, 19% (2019) and 28% (2020) completed the post-encounter assessment, and all students received feedback from faculty and peers. During the standardized patient encounter, students were asked to access and integrate information available in the simulated electronic health record. The students' feedback on the experience was positive, with most of them considering that the standardized patient encounter was extremely or quite effective for practicing skills and strategies necessary for creating the doctor-patient relationship, for interacting with electronic health records, and for verifying electronic data while interacting with patients [33]. Although the authors do not mention the reason for such a low response rate, we believe one reason could be that the survey was voluntary and sent online after the class session.

Similarities/Differences Between Studies With Standardized Patients

From another perspective, some articles included in this study describe different ways of delivering educational content with the help of standardized patients and offer comparisons for unlike methods. One study presents experience with faculty, students, and actors as standardized patients [34]. In this publication, they evaluated the impact of different types of standardized patients on 313 second-year medical students who completed nine-item questionnaires for 908 encounters with faculty, students (peers), and actors as standardized patients. The study results prove that overall, the intervention was effective as a learning experience and was considered valuable and positive, with peers as standardized patients being considered the least valuable. The authors concluded that all types of standardized patients may be useful for educational purposes, with each type having strengths and weaknesses depending on the purpose and the students participating in the teaching experience [34].

Staying on the topic of comparing results using standardized patients and other educational tools, one article questions the means of teaching breast examination to second-year medical students [35]. In this paper, Schubart et al. compared outcomes between 113 second-year students (2008) who were taught clinical breast examination on standardized patients and 131 students (2009) for whom breast simulators were used [35]. All students who participated in the study were tested during their surgical clerkship in their third medical year using simulators, and the results were interpreted based on skills observation and a survey questionnaire. Regarding competence development, the study results demonstrate that using breast simulators yields comparable outcomes to using live standardized patients when teaching clinical breast examination to pre-clinical medical students, with evidence supporting the use of such simulators over the more costly standardized patients [35].

Keeping to the point, one paper compared results using video cases versus standardized patients in teaching second-year medical students problem-based learning. Herein, the authors mark out results obtained after exposing 99 second-year medical students to both video cases and standardized patient encounters and comparing results after all participating students completed a 14-item Likert scale questionnaire. Based on the results obtained, the authors conclude that students found the standardized patient encounters more valuable and believed that using them for problem-based learning was a significantly more positive experience in the following categories: motivation, collaborative learning, reflective thinking, authenticity, doctor-patient communication, and attitude toward patient [36].

Standardized Patient Activities With Combined Teaching Tools

Another study examining methods of delivering educational content describes an intervention that combines simulators and standardized patients for teaching second-year medical students cardiac auscultation. In this research conducted by Friederichs et al., 143 second-year medical students were taught cardiac examination either by using mannequins or by using "hybrid models" with standardized patients wearing the electronic elements of the mannequins used for auscultation. A total of 142 participating students completed a questionnaire based on the different educational approaches. Students found that using "hybrid models" was significantly more well-received than using mannequins, with gratifying results from student tutors and simulated patients [37].

Limitations

Our review was limited because the current search returned a small number of papers for each pre-clinical medical year (14 for first-year medical students, 17 for second-year students, two for both first- and second-year students, one for third-year students, and one not specified). Given that articles found describe educational interventions from various universities across the globe, differences in the medical curriculum must be considered when interpreting results obtained after simulated encounters with standardized patients. The data retrieved and presented comprised solely papers in English, which is an important aspect to consider as a limitation.

Bearing in mind the purpose of this review, we must acknowledge that each study presented had a different method of evaluating results following these sessions and that study heterogeneity is an important limiting factor. In addition, some of the included studies involved small sample sizes, reported low response rates, or were based on older publications, which may further limit the generalizability of the findings.

Another limitation that must be considered is the fact that our search retrieved a small number of studies that followed up on the degree of retainability in the long term.

Future directions

For a better and fuller understanding of the benefits and advantages of standardized patient activities in the curriculum for pre-clinical medical students, more papers presenting long-term retainability of didactic content are needed. In our study, out of 35 articles considered of interest, only four papers followed students on retained skills and student progression after the simulated session, with encouraging results [12,23,27,41].

Furthermore, papers describing the use of standardized patients for other pre-clinical disciplines would be valuable findings for further curriculum development and the use of standardized patients early during medical formation. Our search generated results comprising simulated interventions in the fields of biochemistry [16], virology [14], pharmacology [15], and anatomy [18], as well as interactive didactic sessions on cardiac auscultation [37], breast examination [35], and endocrine physiology [31]. We believe that further expansion of simulated encounters in other disciplines, otherwise restricted to lecture hall teaching, would be an innovative way of delivering educational content.

We believe that standardized methods of evaluation for measuring teaching outcomes are needed when it comes to educational activities with standardized patients.

## Conclusions

We may conclude that this teaching technique is well-received by the pre-clinical students, as they have no contact yet with clinical settings. It may serve as a stepping stone for a better start to clinical education. Students who graduated from these classes might feel more prepared to engage in the hospital environment.

There are potential benefits to using standardized patients as educational tools, even very early in the medical formation years, especially related to the psychosocial aspects of the doctor-patient relationship. This allows novices to practice in a safe environment and teachers to adjust their teaching tools better to meet the needs of medical students. By exposing students to a modern educational environment that focuses on developing soft skills, such educational interventions can improve patient safety and quality of care over time and provide better healthcare outcomes.
